# Risky visuomotor choices during rapid reaching in childhood

**DOI:** 10.1111/desc.12322

**Published:** 2015-07-17

**Authors:** Tessa M. Dekker, Marko Nardini

**Affiliations:** ^1^ Department of Visual NeuroscienceUniversity College London Institute of OphthalmologyUK; ^2^ Department of PsychologyDurham UniversityUK

## Abstract

Many everyday actions are implicit gambles because imprecisions in our visuomotor systems place probabilities on our success or failure. Choosing optimal action strategies involves weighting the costs and gains of potential outcomes by their corresponding probabilities, and requires stable representations of one's own imprecisions. How this ability is acquired during development in childhood when visuomotor skills change drastically is unknown. In a rewarded rapid reaching task, 6‐ to 11‐year‐old children followed ‘risk‐seeking’ strategies leading to overly high point‐loss. Adults' performance, in contrast, was close to optimal. Children's errors were not explained by distorted estimates of value or probability, but may reflect different action selection criteria or immature integration of value and probability information while planning movements. These findings provide a starting point for understanding children's risk‐taking in everyday visuomotor situations when suboptimal choices can be dangerous. Moreover, children's risky visuomotor decisions mirror those reported for non‐motor gambles, raising the possibility that common processes underlie development across decision‐making domains.

## Research highlights

- Comparing participants' choices in a rewarded rapid reaching task with those that would be optimal for maximizing reward, we found that unlike adults, children aged 6 to 11 years followed ‘risk‐seeking’ strategies leading to loss of points.
- Children's errors were not explained by distorted estimates of value or probability, but may reflect changes in action‐selection criteria or protracted development of the ability to integrate value and probability information when planning movements.
- The finding that the developing system favours risky visuomotor choices forms a first step towards understanding how children deal with risk in everyday activities, when suboptimal visuomotor choices can have dangerous consequences.
- Children's risky visuomotor decisions mirror those reported for non‐motor gambles, raising the possibility that common processes underlie development across decision‐making domains.

## Introduction

Whether crossing a busy road, throwing a ball into a basket, or reaching for one cup without knocking another over, humans continuously make risky visuomotor decisions. The best action strategies maximize the probabilities of desirable outcomes while minimizing those of negative ones. For example, we would like to get across the road promptly, but without being hit by a car. Identifying efficient trade‐offs between potential risks and rewards in everyday behaviour can be highly adaptive. Solving this problem is complex, however. It involves correctly judging one's own capabilities – for example, the probability of a reach missing its target – and combining this estimate with information about costs and gains in the environment. Nevertheless, laboratory tasks measuring adults' performance have often found them to be ideal in this respect, maximizing their gains on rewarded tasks by taking into account their own visuomotor capabilities (e.g. Battaglia & Schrater, 2007; Trommershäuser, Gepshtein, Maloney, Landy & Banks, 2005; Trommershäuser, Landy & Maloney, 2006). It is currently not known how and when during life efficient visuomotor decision‐making develops. For a developing system rapidly changing in its physical size, speed, precision, and neural processing (Haywood & Getchell, 2009), it is likely to be especially difficult to form and use stable internal models of the body's visuomotor capabilities to optimize performance. To advance understanding of the development of optimal visuomotor decision‐making during childhood, we measured action choices in 6‐ to 11‐year‐olds and adults during a rewarded rapid reaching task. Participants' actual action choices were compared with those that would achieve the optimal trade‐off between the risks and rewards available in the task.

Visuomotor risk stems from the uncertainty inherent to perception and motor control. For example, a paper ball thrown at a basket may land anywhere along a bivariate probability distribution around the aiming‐point due to noise in location estimates of the arm, hand, ball and basket, and in the neural signals that activate muscles for motor execution (Körding & Wolpert, 2006). Variations in ball end‐points, which depend on the total magnitude of this noise, place a probability on each possible action outcome (i.e. missing or hitting the basket). The formal structure of risky visuomotor tasks can therefore be equated to that of gambling tasks, often used in the field of economic decision‐making (Trommershäuser, Maloney & Landy, 2008). In both types of task, subjects choose between lotteries with given outcome values and probabilities. However, in gambling tasks, the chances of winning are stated *explicitly*, whilst in visuomotor tasks, they are determined *implicitly* by noise (variability) in the sensorimotor system. Like an optimal gambler, an optimal action planner should choose the lottery or action strategy with the largest expected gain.

Trommershäuser, Maloney and Landy (2003a, 2003b, 2008) developed an experimental task that captures the problems faced in everyday visuomotor decision‐making. It allows cost and risk factors to be quantified, and gain‐maximizing choices to be identified. Participants make time‐constrained manual reaches towards a target circle to win points, whilst avoiding a partially overlapping penalty circle that incurs point loss. Because of the time constraint, movements are imprecise, so reaches aimed too close to the penalty circle may accidentally land inside it. To score highly, participants need to shift their reaches some way from the penalty, but not so far that they miss out on potential rewards from the target. For each participant, the optimal (gain‐maximizing) aiming‐point can be calculated and compared to their actual aiming‐point. This predicted optimal aiming‐point depends on each individual's own pointing precision, and varies when penalty values and spatial layouts of the stimulus configuration are altered. Trommershauser *at al*. (2003a, 2003b) showed that adults were able to identify the near optimal solution to this problem and aim for screen locations that maximized expected winnings.

How and when during development are veridical representations of bodily abilities – a prerequisite for identifying gain‐maximizing actions – formed? Infants and children continuously engage in risky visuomotor behaviour and thus have many opportunities to learn how physical limitations affect the results of their actions (Von Hofsten, 2004). Indeed, in their first weeks of walking infants do not discriminate between safe and dangerous slopes and will walk down both, while more experienced 14‐month‐olds will avoid the impossible ones (Adolph, Bertenthal, Boker, Goldfield & Gibson, 1997; Adolph, Tamis‐LeMonda, Ishak, Karasik & Lobo, 2008). Abilities to calibrate actions correctly to objects and affordances continue to develop through childhood. DeLoache, Uttal and Rosengren (2004) showed that 17‐ to 30‐month‐old infants sometimes made serious attempts to perform impossible actions on miniature objects such as trying to climb into a toy car or to put on dolls' clothes. Similarly, infants and even children up to age 7 years attempt to reach towards targets through openings that are too small for their hand (Ishak, Franchak & Adolph, 2014). It is unclear whether selection of inappropriate actions in these studies reflects children's overestimation of their own abilities or differences in how averse they are to failing (e.g. getting stuck). In line with the latter possibility, 17‐month‐old infants attempted to walk through impossibly small openings if the penalty of failing was getting stuck, but became overly conservative if failure meant falling off a table (Franchak & Adolph, 2012). This suggests some knowledge both of own physical capabilities and of ‘costs’ associated with different courses of action in early childhood. Similarly, Bayless and Schlottmann (2010) showed that children aged 5 to 7 years made distorted but sensible judgements about their abilities to roll marbles through goals of different sizes. Moreover, they rated harder marble rolling games with lower prizes as less pleasurable (Bayless & Schlottmann, 2010). By 7 years of age, children thus seem to possess a basic understanding of how a smaller chance of success and a lower reward (or higher penalty) combine to predict a worse outcome.

For making *optimal* visuomotor choices, however, changes in probability and reward must not only be taken into account, but precisely estimated. Optimal (gain‐maximizing) decisions across different conditions in a visuomotor task require precise online estimation of different outcome probabilities (e.g. the chance of missing), and the correct weighting of outcome rewards by these estimates to trade off potential gains and losses. Research on sensory cue integration suggests that it is only at around age 8–10 years that children learn to take their own perceptual uncertainty into account to optimize their perceptual decisions (Gori, Del Viva, Sandini & Burr, 2008; Nardini, Jones, Bedford & Braddick, 2008; Nardini, Bedford & Mareschal, 2010; Petrini, Remark, Smith & Nardini, 2014). It is possible that children also learn to take their own visuomotor certainty into account to optimize their movement decisions around this age. To test this, we employed a child‐friendly version of the rapid‐reaching task developed by Trommershauser *et al*. (2003a, 2003b) in children aged 6 to 10 years and adults. This task allowed us to measure how participants' action choices deviate from those predicted by an optimal action planner that maximizes expected gain. The ways in which performance deviates from these optimal predictions provide clues about the causes of these deviations and the mechanisms that may drive shifts from immature to optimal visuomotor strategy selection.

## Methods

### Participants

Participants were children aged 6 to 11 years and adults: 15 6‐ and 7‐year‐olds (mean age = 7.6, *SD* = 0.3 years, 7 males), 18 8‐ and 9‐year‐olds (mean age = 9.0, *SD* = 0.4 years, 10 males), 15 10‐ and 11‐year‐olds (mean age = 10.8, *SD* = 0.5 years, 7 males), and 15 adults (mean age = 22.4, *SD* = 2.5 years, 7 males). All but three were right‐handed. During training, subjects learned to limit their time‐outs (failures to reach the screen on time) to 5% of the trials or less (see Supplemental Methods). Those who timed‐out more frequently during the main task could be trading off speed for accuracy, potentially leaving more time to make online reach adjustments towards the target. This would confound measures of visuomotor strategy selection with movement corrections *after* the decision process. Therefore, additional subjects with >5% ‘time‐out’ errors (see Procedure) were excluded from the analyses (three adults, three 10‐ and 11‐year‐olds, seven 8‐ and 9‐year‐olds, and six 6‐ and 7‐year‐olds). Some time‐outs were likely caused by occasional lapses in attention rather than trading off speed for accuracy (this would not affect aiming strategy). Because sustained attention develops with age, this probably contributes to higher exclusion rates in younger groups. All participants had normal or corrected‐to‐normal vision and no known neurological or psychological disorders.

### Apparatus

**Figure 1:**
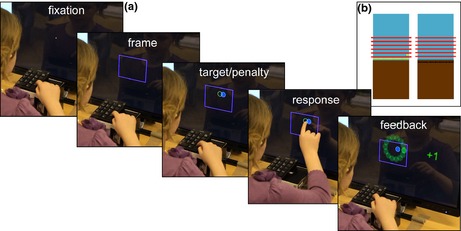
(A) A 9‐year‐old girl demonstrates a typical trial sequence. The blue penalty circle in the example has a gain of −1, and a 1.5 × radius offset from target centre (‘far 1' condition). (B) The current total score (which could be negative) was displayed graphically throughout the session. The brown area is ‘the ground’, the blue area ‘the sky’. For each point gained, a coin appeared (left example). For each point lost, a coin was removed. For every 20 coins (denoted by a red line), subjects won a token that could be exchanged for toys (or £0.50 for adults) at the end. If the total score became negative, each lost point would create a ‘hole’ in the ground (right example). Before getting back into the sky, coins had to be won to fill up the holes.

Participants sat in front of a 24‐inch monitor with integrated touch sensors (Iiyama ProLite T2451MTS MultiTouch screen, with 521 × 293 mm display area and 1920 × 1080 resolution), at 26 cm distance for adults, 20 cm for children. A wireless numeric keypad was mounted on the table, centred before the monitor 6 cm from the participant's trunk (see Figure 1A). Tasks were run using Matlab 7.1 (R2010a) with Psychophysics Toolbox (Brainard, 1997).

### Design and stimuli

Stimulus configurations consisted of a target circle with a green outline and a partially overlapping penalty circle, each with a 0.90 cm (33 pixel) radius. Target circles were unfilled, while penalty circles were filled with a darker shade of their yellow, blue or grey outline colour (see Figure 1A). A blue 114.2 × 80.6 mm frame at the screen centre indicated where the circles would appear. To prevent use of pre‐planned movements, on each trial the whole stimulus configuration was ‘jittered’ around the frame's centre, with horizontal and vertical offsets drawn independently from a uniform ±44 mm distribution. We varied whether the horizontally displaced penalty circle was located near to or far from the reward (1 versus 1.5 circle‐radius displacement), and whether hitting it incurred a small or large point loss (1 versus 5 points), resulting in four conditions: ‘near 1’, ‘far 1’, ‘near 5’, and ‘far 5’. For half of each age group the low penalty was presented for the first half of the task, and for the others the high penalty was first. Distinct penalty colours signified different values; blue for 1, yellow for 5 and grey for 0 in training. Near and far offsets were presented equally often in random order within blocks. To keep trial numbers to a child‐friendly minimum, the left / right positioning of circles was counterbalanced across participants. Half in each age group had far penalty offsets on the left and near offsets on the right; for the others these positions were reversed.

### Procedure

#### Training phase

Time limits ensured that subjects made ballistic reaching movements without online corrections. To allow for individual differences in reach reaction times, a pre‐experimental training phase included a selection procedure that established individual time limits. In 13–15 blocks of 20 trials, participants attempted to hit targets under time limits that decreased when they performed to criterion (see Supplemental Methods). Training trials were identical to the experimental trials described below, except that target hits did not count towards the final score, and the (grey) penalty circle was irrelevant.

#### Experimental phase

Each training trial started with a central fixation cross. Participants pressed the ‘Enter’ key with the index finger of their dominant hand to trigger presentation of the blue frame (see Figure 1A). The stimulus configuration appeared inside the frame after 500 ms of pressing. Participants then released the key and touched the screen with the same finger. If they released less than 100 ms after target onset (anticipation) the trial was aborted. Touching the target circle within the time limit gave a reward of 1 point. Touching the penalty within the time limit incurred a loss of 1 or 5 points. For the overlapping region of penalty and target, the reward and penalty were summed. A touch anywhere else on the screen was a ‘miss’ (no gain or loss). A ‘time‐out’ incurred a 7‐point loss and a waiting period of 20 seconds. Participants were simply instructed to obtain as many points as possible.

There were 10 blocks of 20 trials, a total of 50 per condition. Penalties and payoffs were explained before the first and sixth block when penalty rules changed. Before each block, participants were asked to repeat these back to ensure that all remembered the current outcome values. Running scores were displayed graphically in a chart on the screen (Figure 1B). As a motivation for maximizing their points, participants received a prize token for every 20 points they earned. At the end of the session, each token was converted to £0.50 for adults or exchanged for small prizes for children. Subjects also performed three additional tasks that measured their understanding of the point system of the experiment (*number line and multiplication task*), and their explicit knowledge of their own visuomotor ability (*hit probability task*).

#### Number line task (measuring value distortion)

All subjects took part in a pencil‐and‐paper number line task. On each trial, subjects used a pencil to indicate, with a line, where on a bar of length 10 cm a number would fall. The left and right ends of the bar were labelled 0 and 10, respectively, and the numbers 1–5 were tested 10 times each, with numbers randomized within each block. The marked locations were measured and compared with the locations predicted by a linear representation of the number line (e.g. the number 4 would fall at 0.4 of the length of the 10 cm line, at 4 cm).

#### Multiplication task (measuring value distortion)

All subjects took part in a two‐alternative forced choice task with 35 trials. On each trial they chose between a pair of values (7 repetitions of 5 pairs). Each pair consisted of the standard value of ‘5 points × 1’, and a comparison in which ‘1 point’ was multiplied with the values 3 to 7 (‘1 point × 3’, ‘1 point × 4’, etc.). For each pair, subjects were asked to select the value *they would prefer to lose* (the correct answer was the lower value). For example: would you prefer to lose 5 points once (5 × 1), or to lose 1 point four times (1 × 4)? The task was described in terms of losses to best match the penalty situation in the main experiment. To make the problem concrete, subjects received 10 stickers or snacks and were informed that one of their answers, randomly drawn at the end of the task, would determine how many they would have to give back. For each age group and comparison value, we counted how often the constant sum was chosen over the variable sum. We used the standard psychophysical method of fitting a cumulative Gaussian distribution through the resulting proportions (using the Psignifit toolbox for Matlab), and determining the Point of Subjective Equality (PSE); see Figure 6B. The PSE is the point along the *x*‐axis at which the comparison and standard are chosen with equal (0.5) probability, and therefore measures the point at which they are, on average, judged to be equal. Deviations of the PSE from the correct value of 5 (1 × 5) thus provide information about systematic under‐ or overestimations of the value of losing 5 points relative to the value of losing 1 point multiple times.

#### Hit probability task (measuring probability distortion)

In a hit probability task, subjects were presented with five circle sizes in a display such as the one used in the experiment. In 10 blocks, all circles were presented in random order (50 repetitions in total). The circles were presented in isolation (without an overlapping penalty circle), and their locations were jittered as in the experiment. Circle sizes were scaled individually for each subject based on their own visuomotor variance measured during the main task. The five circle sizes seen by each subject corresponded to hit probabilities of 0.1, 0.3, 0.5, 0.7, and 0.9. On each trial, subjects judged ‘how many times out of 10’ they would successfully hit the circle, from the same position and with the same time constraint as in the main experiment. This provided their estimated probability of hitting (e.g. ‘5 out of 10’ = 0.5). Judged probabilities were averaged over 10 repetitions (50 trials in total). Mean judged probabilities were averaged across all subjects in an age group, and compared with the true probabilities (95% error bars).

### Analysis

#### Measures

On each trial, reaction time (target onset to screen touch), touched horizontal (X) and vertical (Y) screen position, and score were recorded. ‘Time‐out’ trials were excluded from the analysis (see Table 1 for details). For model predictions (see below), the scatter of movement end‐points was fitted by a bivariate Gaussian distribution. Outlying data points can strongly influence these distributions and model predictions. Outliers were therefore excluded using the Minimum Covariance Determination (MCD) procedure (Rousseeuw, 1984) (~5% of trials excluded in all age groups); see Supplemental Material for details. Mean reaction time, aiming‐point (*mean X*,* mean Y*), horizontal and vertical variance (*varX* and *varY*), and total score were computed for each subject and condition using the remaining data.

**Table 1:** Descriptive experimental data displayed per age group. Stars in adult column indicate that age difference across all groups is significant at p < .05

	Adult *N* = 15 age = 22.4 (2.5)	10–11 *N* = 15 age = 10.8 (0.47)	8–9 *N* = 18 age = 9.0 (0.35)	6–7 *N* = 15 age = 7.6 (0.34)
Time limit (secs)				
Mean	0.66 (0.04)*	0.75 (0.06)	0.78 (0.05)	0.83 (0.04)
Range	0.60 – 0.75	0.65 – 0.85	0.70 – 0.85	0.75 – 0.9
No. time outs	4 (2.2)*	7.2 (2.1)	5.3 (2.8)	6.5 (2.6)
Reaction time (secs)	0.53 (0.04)*	0.59 (0.05)	0.61 (0.04)	0.64 (0.05)
Vertical aim points (cm)	−0.03 (0.1)*	−0.12 (0.1)	−0.06 (0.1)	−0.006 (0.1)
Deviation from X_mg_ (cm)	0.11 (0.07)	0.18 (0.1)	0.16 (0.1)	0.13 (0.1)
Variance (cm)				
Horizontal	2.44 (0.82)*	2.51 (0.93)	2.96 (1.3)	3.69 (1.4)
Vertical	4.11 (1.51)*	3.31 (1.25)	4.21 (2.08)	5.05 (2.27)

#### Model predictions

**Figure 2:**
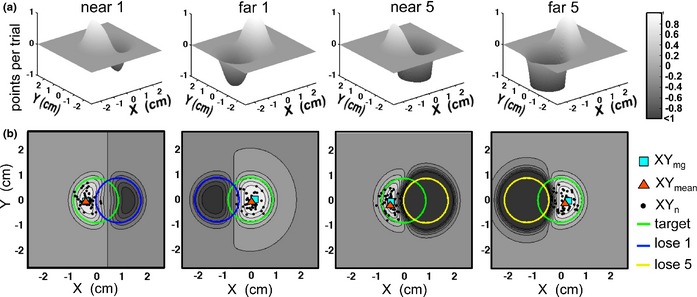
(A) Gain landscapes from example subject presented with ‘near’ targets on the left and ‘far’ targets on the right. These plots show the raw data as collected. For further analysis, X‐coordinates were flipped into a common orientation so that shifts from the target centre away from the penalty area were always positive. For each condition, expected points per trial are displayed for each possible aiming location in a 200‐pixel square around the centre of the target circle. The X‐coordinate with the highest expected gain is at the peak of each landscape. (B) Contour plots of the same data, with overlaid stimuli (large circles). A blue square indicates the peak of each gain landscape. Movement end‐points on individual trials are plotted as black dots, and their means are indicated by red triangles. This subject's mean aiming‐points fell close to gain‐maximizing coordinates (blue circles) for all conditions, showing that their visuomotor strategies were near‐optimal.

The aiming location *X*_*mg*_,*Y*_*mg*_ that would maximize expected gain given that the visuomotor precision *varX*,* varY* was computed for each subject and condition following the ideal actor / observer model described in Trommershauser *et al*. (2003a, 2003b). 4 × 4 (Age × Condition) ANOVAs revealed that there were no significant differences in X or Y variance across conditions, a pattern consistent across age groups (largest *F* for Condition and Condition × Age <1.62, *p* = .194). Variance estimates for each subject were therefore obtained by pooling data across conditions. The expected consequences of aiming towards any given location on the screen were modelled as depending on a bivariate Gaussian distribution described by each participant's own X and Y variance. The gain expected from pointing at any location is given by a weighted sum of all the possible outcomes, in which each outcome (e.g. +1, −5, 0) is multiplied by the probability of a reach landing in a location with this outcome. For each participant and condition, we computed the ‘gain landscape’ describing the gain expected from aiming for each pixel in a 200 × 200 grid around the target centre (Figure 2); see the Supplemental Material for details. The optimal aiming coordinate *X*_*mg*_,*Y*_*mg*_ is at the peak (maximum) of this landscape (Figure 2A, and Figure 2B, cyan squares), and its height represents the optimal‐predicted average gain. In Figure 2B, each condition's gain landscape is overlaid with the stimulus configuration (circles), and the empirical distribution of movement end‐points from the same example adult participant whose X and Y variance was used to calculate the landscape. In this participant, the optimal prediction (cyan circle) changes across conditions, and the mean of the empirical points (red triangle) follows it closely.

## Results

### Reaction times and end‐point variances

Reaction times decreased significantly with age, as revealed by Age × Condition ANOVAs (Age: *F*(3, 59) = 30.7, *p* < .01) and in accordance with age‐related shortening of individual time‐limits (Age: *F*(3, 59) = 13.6, *p* < .01; see Table 1). Age × Condition ANOVAs showed that horizontal and vertical variance also varied with age (X: *F*(3, 59) = 5.77, *p* = .002; Y: *F*(3, 59) = 3.58, *p* = .019), with significantly larger variances than adults only for 6‐ to 7‐year‐old children for both X and Y (*p* < .05 in Sidak‐corrected post‐hoc comparisons; see Table 1).

### Do vertical aiming‐points maximize expected gain?

The Y‐coordinate with the highest predicted score fell at the centre of the stimulus configuration (Y_0_) for all conditions. Accordingly, the height of subjects' aiming‐points did not vary by condition (Condition: *F*(3, 57) = 1.62, *p* = .19; Condition × Age: *F*(9, 17) = 1.43, *p* = .18). Aiming‐points did, however, vary significantly with Age (F(3, 59) = 3.58, *p* = .019); see Table 1. Post‐hoc tests revealed that while adults and 6‐ and 7‐year‐olds had mean Y‐coordinates indistinguishable from the optimal Y_0_ (adults: *t*(14) = −1.44, 6–7: *t*(14) = −0.33), both *p*‐values > .1), 8‐ and 9‐year‐olds and 10‐ and 11‐year‐olds tended to aim slightly below this point (10–11: *t*(14) = −8.4, *p* < .001; 8–9: *t*(17) = −2.6, *p* = .02).

### Do horizontal aiming‐points maximize expected gain?

#### Adults

**Figure 3:**
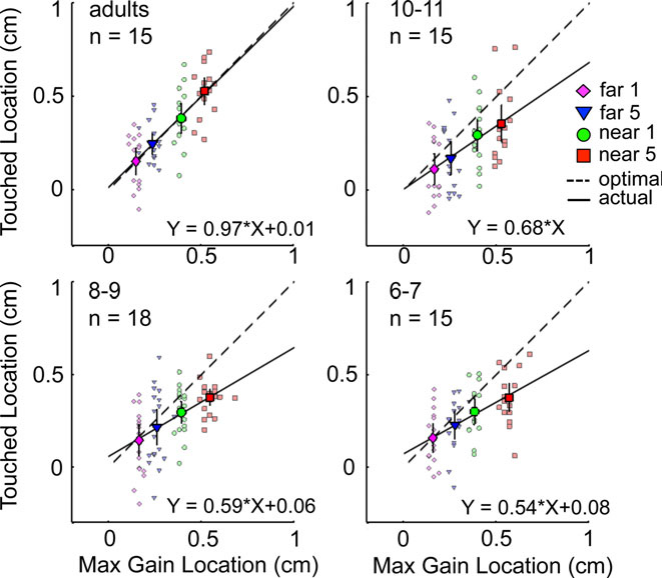
Empirical mean X‐coordinates (Touched Locations) are plotted against the predicted gain‐maximizing X‐coordinates (Max Gain Locations) of each condition and subject in each age group. Condition means (95% CI error bars) are indicated by larger symbols. For points falling along the dashed identity line (intercept 0, slope 1), the touched and max gain location were identical, so visuomotor strategies were optimal. Points below the line are those of subjects who did not place their aiming‐points far enough from the penalty circle to maximize their expected gain; those above the line were placed too far. Regression lines were fitted to all data points to visualize differences in movement strategies across age. Slopes smaller than 1 reflect a tendency to undershoot the optimal aiming location.

To test whether adults were able to find their own gain‐maximizing coordinate by weighting outcome values by their probabilities, we compared their actual and predicted optimal aiming‐points (Figure 3). A linear regression (*R* ^2^ = 0.523, *F*(1, 58) = 63.51, *p* < .001) found a slope of 0.97 and intercept of 0.01 (Figure 3, dashed line). These regression parameters closely match those of the identity line with slope 1 and intercept 0. Adults' mean X‐coordinates thus fell on average extremely close to the corresponding predicted gain‐maximizing coordinates. To find this coordinate in each condition's gain landscape, subjects should shift their horizontal aiming‐points further from the target centre when penalty values increase and when penalty and target circles are closer together. A 2 × 2 (Value × Offset) ANOVA revealed that adults indeed made these adjustments across conditions (Value: *F*(1, 14) = 20.45, *p* < .001; Offset: *F*(1, 14) = 22.7, *p* < .001; interaction not significant, *F*(1, 14) = 2.52, *p* = .134). Moreover, adults shifted by the correct distance to maximize their expected gain given their own visuomotor precision; an ANOVA comparing actual and optimal aiming‐points across the four conditions showed that adults' mean X‐coordinates were statistically indistinguishable from their gain‐maximizing X‐coordinates (main effect of actual‐optimal, *F*(1, 14) = 0.024, *p* = .88; actual‐optimal × condition interaction: *F*(1.55, 21.7) = 0.058, *p* = .91).

#### Children

With age, movement end‐points shifted closer towards the gain‐maximizing X‐coordinates, and regression slopes for optimal vs. actual points (Figure 3) clearly became steeper and closer to the identity line. Means (large circles) below the identity line indicate that children's aiming‐points were on average placed too close to the penalty circle to land on the gain‐maximizing coordinate.

A three‐way Actual‐Optimal × Condition × Age ANOVA confirmed that distances between actual and optimal aiming‐points become smaller with age from childhood to adulthood (main effect of Actual‐Optimal: *F*(1, 59) = 54.38, *p* < .001; Actual‐Optimal × Age interaction: *F*(3, 59) = 6.68, *p* = .001). Distances between actual and optimal aiming‐points also varied with condition; they were larger in those conditions for which the gain‐maximizing coordinate required a larger shift from the target centre; see Figure 3 (Actual‐Optimal × Condition interaction: *F*(1.4, 80.1) = 5.9, *p* = .01; this effect did not depend on age: *F*(4.1, 80.1) = 0.78, *p* = .54). Follow‐up ANOVAs revealed that even by ages 10 and 11 years, children's mean X‐coordinates still significantly undershot the predicted gain‐maximizing location (Actual‐Optimal × Condition interaction at 10–11 years: *F*(1, 14) = 29.28, *p* < .001; 8–9 years: *F*(1, 17) = 18.23, *p* = .001; 6–7 years: *F*(1, 14) = 44.15, *p* < .001). Thus, even in relatively late childhood, movement strategies were suboptimal, and carried unnecessarily high risks of touching the penalty.

While children up to age 10 and 11 years systematically mis‐localized the gain‐maximizing coordinate, some sensitivity to gain landscape changes across conditions was already evident by 6 and 7 years. As Figure 3 shows, the order of condition means along the *y*‐axis is consistent across groups, so, like adults, children shifted their aims away from the target centre as a function of both penalty value and offset. ANOVAs found significant effects of both factors at all ages: for Value, 10–11 years: *F*(1, 14) = 7.68, *p* = .02; 8–9 years: *F*(1, 17) = 23.61, *p* < .01; 6–7 years: *F*(1, 14) = 5.15, *p* = .04; for *Offset*, 10–11 years: *F*(1, 14) = 5.53, *p* = .03; 8–9 years: *F*(1, 17) = 6.87, *p* = .02; 6–7 years: *F*(1, 14) = 5.63, *p* = .03); no interaction in any group, largest *F* = 0.20, *p* = .63. The deviation between the optimal and actual aiming‐point in childhood did not significantly depend on whether the low or high penalty was presented first (2 × 2 ANOVAs for Penalty Order × Actual vs. Optimal X‐coordinate: ‘near 1’: *F*(1, 42) = 3.49, *p* = .069, ‘far 1’: *F*(1, 42) = 0.79, *p* = .38, ‘near 5’: *F*(1, 42) = 0.001, *p* = .97 ‘far 5’: *F*(1, 42) = 0.19, *p* = .67). It is therefore unlikely that these sub‐optimal aiming strategies were due to rule switching difficulties when penalties changed halfway through the task.

### Computing versus learning the best action strategy

Gain‐maximizing coordinates can be computed directly by weighting outcome values by their visuomotor noise‐dependent probabilities to estimate the peak of the gain landscape (see Figure 2). However, they can also be found by searching for aiming‐points with higher rewards (reinforcement learning). Regression lines fitted across the trial time series of each condition for each individual subject revealed remarkably consistent aiming strategies throughout the task (one‐sample *t*‐tests of slope versus 0: All *p*‐values > .05, with Sidak‐correction for 252 comparisons; except for one 10‐ to 11‐year‐old in the ‘near 1’ condition). So, in line with previous findings (Trommershäuser *et al*., 2003a, 2003b), both adults and children seem to select their action strategy early on without apparent learning.

### Effects of movement strategies on scores

**Figure 4:**
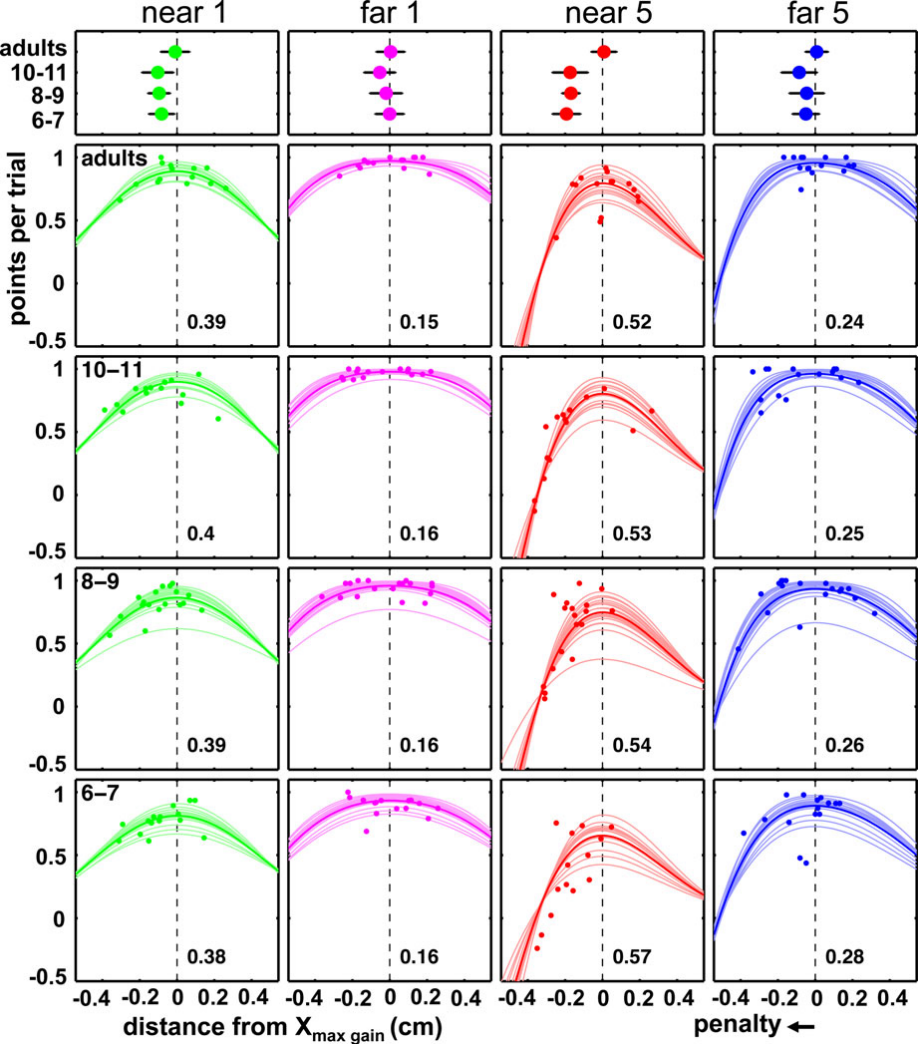
Top row: mean deviations from the gain‐maximizing X‐coordinate (set to x = 0, indicated by dotted lines). Error bars are 95% confidence intervals. Positive (right) and negative (left) deviations reflect, respectively, mean aiming‐points falling too far away from, or too close to, the penalty area to land on the gain‐maximizing coordinate. Bottom four rows: curves plot predicted scores for each subject across aiming‐points deviating relative to the optimal aiming location (dotted line, 0 deviation). Numbers next to dotted lines are the mean optimal aiming‐point (in cm, with respect to the target centre) for the age group and condition. Scattered data points are individual subjects' actual deviations from X_maxgain_ plotted against their actual obtained points per trial.

Subjects were not explicitly instructed to aim for gain‐maximizing coordinates, but only to score as many points as possible. A crucial question, therefore, is whether children's deviations from gain‐maximizing X‐coordinates did indeed result in reduced scores. Otherwise, children might have had little reason to choose closer‐to‐optimal strategies. Solid curves in Figure 4 display for each subject and condition how predicted scores decrease as aiming‐points depart from the gain‐ maximizing coordinate (*X*_*mg*_, dashed line, at zero on the *x*‐axis). The data points plot distances between participants' actual and gain‐maximizing X‐coordinates against their obtained rewards (mean points per trial). Obtained and model‐predicted scores are likely to differ somewhat due to noise, since movement end‐points are ‘sampled’ probabilistically from a subject's visuomotor distribution. However, most data points fall within the range of the predicted curves, revealing a correspondence between model predictions and task performance.

Expected gain curves are asymmetrical, with lower predicted scores for movement end‐points undershooting the gain‐maximizing coordinate (left of the peak) than for those shifting too far (right of the peak) by the same distance. In childhood (three bottom rows), many end‐points fall left of the peak (closer to the penalty), at X‐coordinates with relatively lower predicted scores. Obtained scores are accordingly lower. In contrast, adult movement end‐points (second row in Figure 4) are clustered around the peaks of the gain landscapes, along aiming locations with high associated gain. Note, however, that scores are not only determined by whether movement end‐points undershoot or overshoot the peak on average (each group's bias), but also by how this varies across individuals (each group's variance). These deviations from the optimal aiming‐point are captured by the points and error bars in Figure 4 (top panel), the spread of data points in Figures 3 and 4, and the absolute deviation from X_mg_ in Table 1. The spread of end‐points in adults reveals that even some mature subjects missed their optimal coordinate by some distance. Indeed, mean absolute deviations from the gain‐maximizing location in Table 1 are smallest in adults, but only slightly smaller than in children. Consequently, even adults may not score 100% of their predicted maximum on average. Tukey's post‐hoc tests revealed that adults have smaller mean absolute deviations than 8‐ and 9‐ and 10‐ and 11‐year‐olds (*p* = .35 and .01, respectively), but not than 6‐ and 7‐year‐olds (*p* = .42). Therefore, any differences in score between the oldest and youngest groups will be mainly due to a different directional bias in movement selection (i.e. aiming too close to penalty at ages 6–7).

**Figure 5:**
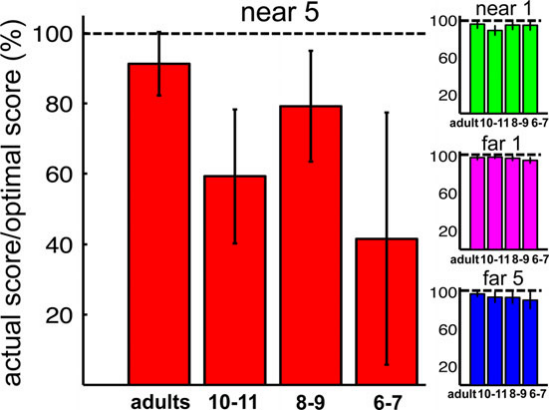
Mean (95% CI bars) of individuals' actual scores expressed as a percentage of their own highest expected (optimally‐predicted) scores. At 100%, obtained and highest expected scores are identical.

To test whether age groups differed significantly in their abilities to maximize gain, scores were expressed as percentages of the predicted maximum score (Figure 5) and compared in a two‐way ANOVA (Condition × Age). In line with the more sub‐optimal aiming‐points selected at younger ages, scoring efficiency decreased significantly with age (*F*(3, 59) = 5.07, *p* = .003), but this effect was modulated by condition (Age × Condition: *F*(3.8, 73.9) = 2.91, *p* = .03). Follow‐up ANOVAs showed that scores only significantly differed by age in the ‘near 5’ condition with the steepest gain landscape; ‘near 5’ condition: *F*(3, 59) = 3.78, *p* = .015, all other conditions: largest *F* < 1.44, *p* = .24). In the ‘near 5’ condition, all three child groups scored significantly below their optimal predictions (10–11 years: *t*(14) = −4.20, *p* = .01 8–9 years: *t*(17) = −2.56, *p* = .02, 6–7 years: *t*(14) = −3.20, *p* = .006). Adults showed a non‐significant trend towards a sub‐optimal mean score in this condition (*t*(14) = −1.89, *p* = .08).

In sum, these findings clearly show that children aged 6 to 11 years chose reaching strategies with an overly high risk of hitting the penalty circle, and that this had detrimental effects on the rewards they obtained. For example, 6‐ and 7‐year‐olds won less than 50% of their potential points in the most challenging condition (Figure 5). Additional control tasks were conducted to understand *why* children aimed so ‘dangerously’ close to the penalty area (a risk‐seeking strategy).

### Do younger subjects distort outcome values?

**Figure 6:**
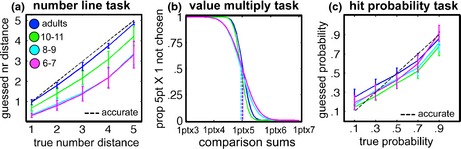
(A) Mean (95 CI error bars) estimated numerical distance on number line is plotted against true numerical distance for each group. The dashed identity line indicates accurate numerical distance representation. Data points under this line reflect underestimation of true numerical distance. (B) Mean proportion of times the variable sum (x‐axis) was preferred over the standard sum (5pts × 1) when asked ‘which amount would you rather lose?’ Cumulative Gaussians are fitted through the data points of each group. Points of subjective equality (dashed lines) measure how many multiples of 1 subjects were as willing to lose as a single 5. (C) For each group, mean (95 CI error bars) estimated probabilities of hitting circles of different sizes are plotted against their true probabilities of hitting these circles. The dashed identity line indicates accurate probability estimates. Data points under this line reflect underestimation of own precision, and points above the line overestimation.

Young children often show nonlinearities in their understanding of number, typically underestimating larger values (Booth & Siegler, 2006). In the reaching task, this could lead to underestimation of the severity of larger penalties (5 points) and to subsequent underestimation of the shift in pointing required to maximize expected gain – the reaching pattern found in childhood. To test whether value distortion explained children's reaching behaviour, two measures of value representation were obtained. In a number line task, subjects indicated where the numbers 1 to 5 belonged on a number line between 0 and 10; see Supplemental Methods for details. Younger subjects' responses were compressed towards the lower end of the scale, indicating underestimation of numerical distance (Figure 6A). Accordingly, the root of the mean squared error (RMSE) of indicated versus true numerical distance became smaller with age (*r* = −0.60, *p* < .001). However, this summary measure of numerical distance distortion did not predict subjects' visuomotor strategies: the correlation of RMSE with absolute deviation between *X*_*mean*_ and *X*_*opt*_ was not significant for any single condition, or for the mean of all conditions (*r* < 0.09, *p* < .47), and hence did not explain performance on the reaching task.

Correct value comparisons between gains and losses crucially relied on understanding the relative value of quantities 1 and 5. Therefore, in a multiplication task, subjects judged whether they would rather lose 5 points once, or 1 point multiple times (e.g. 1 point, 6 times); see Supplemental Methods for details. Cumulative Gaussians were fitted to proportions of trials on which participants preferred to lose the variable multiple (e.g. 1 point, 6 times) over losing 5 points; Figure 6B. The point of subjective equality (PSEs) of these functions, at which the probability of answering either way is 0.5, measures the multiple of 1 that participants are as willing to lose as a single 5. The PSE in Figure 6B falls very close to the correct multiple of 5 for all age groups (adults: 5.02, 10–11: 4.95, 8–9: 4.95, 6–7: 5.00), revealing that the impact of losing 5 points compared to winning multiples of 1 point was judged close to accurately at all ages. Both tasks thus suggest that point value distortions are an unlikely cause of the sub‐optimal reaching choices in childhood.

### Do younger subjects distort outcome probabilities?

Forming stable representations of developing visuomotor skills might be challenging; if younger subjects distorted outcome probabilities by underestimating their own pointing variance *varX*, this would lead them to underestimate the shift from penalty required to maximize their winnings. To test whether probability distortions explained children's tendency to undershoot the gain‐maximizing coordinate, understanding of own visuomotor precision was measured after the main experiment. Participants gave verbal estimates of how many times out of 10 they could hit target circles of different sizes with the same setup and time limit as in the reaching task; see Supplemental Methods for details. In Figure 6C, mean judged probabilities are plotted against true probabilities of hitting each circle. As visualized in the graph, explicit judgements of visuomotor skill were on average close to accurate at all ages, with slight tendencies to overestimate small probabilities and to underestimate large probabilities. Importantly, younger subjects did not overestimate their chances of hitting target circles compared with adults, but showed similar probability distortions. This is in line with other findings of adult‐like event probability judgements in childhood (Boyer, 2006; Moutsiana, Garrett, Clarke, Lotto, Blakemore *et al*., 2013). There was no evidence for overly high confidence in own visuomotor precision in childhood, suggesting that this factor cannot explain the development of optimal visuomotor decision‐making.

## Discussion

To advance understanding of when and how optimal visuomotor decision‐making develops in childhood, 6‐ to 11‐year‐olds and adults were asked to rapidly reach towards targets to win as many points as possible, whilst avoiding touching an overlapping penalty area. Because time was limited, movement end‐points were imprecise, placing probabilities on missing or hitting the target and penalty. The implicit task was to locate the aiming‐point giving the highest expected score by accounting for this imprecision. By quizzing subjects about the point rules between blocks, we ensured that all could remember these throughout the experiment. To maximize their expected winnings, subjects should shift their aiming‐points further from the penalty area when penalties were greater and closer to the target. The precise amount of shift required to optimize performance depended on each participant's own visuomotor precision.

Sensitivity to changes in gain landscapes across conditions was already present at 6 and 7 years; all age groups adjusted their movement end‐points in response to changes in both penalty value and offset. However, the ability to correctly determine the exact degree of adjustment required to maximize expected gain, depending on these factors *and* on participants' own visuomotor precision, did not develop until much later. Unlike adults, even the oldest (10‐ and 11‐year‐old) group of children still differed significantly from optimal. Children of all ages displayed a group‐level tendency to place movement end‐points too close to the penalty circle to maximize their scores (see Figure 3), resulting in larger deviations from the gain‐maximizing strategy in childhood. Choosing end‐points too close to the penalty region can be described as risk‐seeking because this comes with overly high risks of hitting the penalty circle and losing points. This sub‐optimal strategy had a dramatic impact on the rewards won in the study, particularly in the ‘near 5’ condition, which presented the steepest gain landscape (Figure 4). Here children's systematic ‘undershoot’ incurred a heavy penalty (Figure 4); unlike adults, who obtained on average >90% of their theoretical maximum score, 6‐ and 7‐year‐old children obtained as little as ~40% (Figure 5).

What can explain the shift from overly risky visuomotor decision‐making in childhood to close to optimal visuomotor decision‐making in adulthood? The most punishing condition (near 5) only comprised 25% of the trials. Did children stick with risk‐seeking strategies because they were not sufficiently motivated to optimize their scores? This is not supported by the data; ANOVAs revealed that children were sufficiently motivated to change their aiming strategy across conditions – except that they systematically placed their new aiming‐points too close to the penalty area. Further analyses revealed that this was not due to difficulties with switching between penalty rules or slower learning of where in the display most points could be won at younger ages. The patterns of children's errors could reflect specific distortions in estimates of outcome values or probabilities (underestimation and overestimation, respectively). However, control tasks measuring value representation and understanding of own visuomotor precision (Figure 6A–C) provided no evidence for such distortions. While more research is needed to investigate how children represent the outcomes of their actions and the associated probabilities, these measures strongly suggest that their suboptimal visuomotor decisions in this reaching task were not driven by inaccurate value or probability estimates. Children's risky aiming strategies therefore likely reflect immature decision‐making processes.

This leaves us with two interesting explanations. Firstly, sub‐optimal reaching might result from incorrect computation of the optimal strategy by weighting of outcome values by probabilities. Immature weighted averaging would be in line with recent developmental studies showing that abilities to average multiple sensory estimates to make optimal perceptual decisions are still developing until the 8th–11th year of life (Gori *et al*., 2008; Nardini *et al*., 2010). Those results come from un‐rewarded psychophysical tasks, and the present findings raise the possibility that mechanisms driving this development may also affect visuomotor processing in relation to external cost factors.

Alternatively, children might be able to compute the gain‐maximizing strategy, but not employ this information to optimize performance. Instead, they might pursue goals other than maximizing gain, such as striving for variation in the results of their reaches or fulfilling a desire to hit the target as often as possible, irrespective of overall winnings. Both could lead to avoidance of more conservative strategies with higher score expectations (for similar explanations of risk‐taking in rhesus monkeys, see Hayden, Heilbronner, Nair & Platt, 2008). Preferring risky actions could be adaptive for a developing system, in creating more opportunities to learn about consequences of behaviour (Schneider, Hanne & Lehmann, 1989). Children's visuomotor strategies could thus be gain‐maximizing in the long run if the future gain provided by learning outweighs the rewards lost during the experimental task.

To better understand the underlying representations and mechanisms, future studies should compare how the expected gain of an embodied gamble is represented in the developing brain. In adults, for example, increasing the expected value of a ‘classic’ gamble yields parametric activation increases in brain regions associated with reward and decision‐making, such as the ventral striatum and orbitofrontal cortex (e.g. Peters & Büchel, 2010; Rolls, McCabe & Redoute, 2008). In adolescents, some of these areas are overly responsive to expected gain in line with their behavioural preference for risky gambles (Barkley‐Levenson & Galván, 2014). If children are still developing the ability to combine visuomotor and cost information into an accurate expected gain estimate, similar age differences should be present in brain areas representing the expected gain of a visuomotor choice.

This work significantly extends findings from other decision‐making domains. It was recently proposed that classical and visuomotor decision‐making rely on different neural mechanisms because adults show near‐optimal performance in the visuomotor domain but display well‐known value and probability distortions when making equivalent choices in a gambling task (Wu, Delgado & Maloney, 2009). This dissociation has been called into question, however, and differences in performance have instead been ascribed to differences in task (Jarvstad, Hahn, Rushton & Warren, 2013). In line with current findings in the visuomotor domain, children's choices during classic gambles have also been characterized as risk‐seeking (Harbaugh, Krause & Vesterlund, 2002; Levin, Hart, Weller & Harshman, 2007; Paulsen, Platt, Huettel & Brannon, 2011). This raises the intriguing possibility of a common developmental mechanism configuring both classic and visuomotor decision‐making. If development were indeed linked across domains, this would provide strong evidence for a shared underlying process. An important next step would therefore be to directly compare the development of classic and visuomotor gambling within subjects using well‐matched tasks.

In sum, we found a clear, age‐related shift towards more optimal visuomotor decision‐making across childhood and adulthood, with overly risk‐seeking action selection between ages 6 and 11 years. Choosing actions with overly high risks of negative results in real‐life visuomotor tasks can clearly be dangerous, and could be a factor significantly contributing to higher accident rates in childhood (Kingma, 1994; Sethi, 2008).

## Supporting information


**Data S1.** Supplementary Methods.
