# Supplementary material for: Risky visuomotor choices during rapid reaching in childhood
Source: Dev Sci. 2015 Jul 17;19(3):427–39. doi: 10.1111/desc.12322 (PMC4975720; doi:10.1111/desc.12322)
Supplement: Supplementary file 1 — Data S1. Supplementary Methods. [file DESC-19-427-s001.docx]

**Supplementary Methods**

*Time limit selection procedure during practice*

After an initial block allowing unlimited time, participants were introduced to a time limit of 1 second for the following block. In this and in subsequent training blocks, participants were encouraged to hit targets within the time limit. Following a block in which subjects attained a 90% (18/20) or better hit rate, and did not “time out” (exceed the time limit), the time limit decreased; first to 0.9 and then further in 0.05-second intervals. Otherwise, the same time was repeated for the next block. The time limit reached two blocks before the end was repeated for the final two training blocks and used in the experiment. In total, there were 13 blocks for adults and 15 blocks for children.

*Removal of outliers*

The FAST-MCD algorithm (Rousseeuw & Driessen, 1999) as implemented in the Libra toolbox for Matlab (Verboven & Hubert, 2005) was used to remove outliers from the response distribution. Specifically, the “robust” Mahalanobis distance of each data point was computed based on robust estimates of the mean and covariance matrix (assuming 1% of aberrant responses; α = 0.99), and significant outliers (exceeding a cut-off distance of = 2.72) were identified for exclusion. Percentage of outliers adults: 5.1% (SD: 1.1%), 10-11 years: 5.1% (SD: 1.2%), 8-9 years: 5.3% (SD: 1.1%), 6-7 years: 5% (SD: 1%).

*Computing individual gain landscapes*

The probability of hitting a circular area with radius R when aiming at point *X_p_Y_q_* on the screen with distance *D* from the circle centre can be calculated by centring the subject’s bivariate Gaussian response distribution on the *X_p_Y_q_* coordinate, and calculating the area under the distribution overlapping with the circle. The integral of the response distribution across the penalty circle and target circle was thus computed for each possible aiming point *X_p_Y_q_* inside a 200x200 pixel square around the target centre. The aiming point *X_mg_Y_mg_* that maximizes expected gain for a given condition is the point with the highest expected score per trial, given by *P_target_ * gain target + P_penalty_ * gain penalty*. Expected point loss due to timing out can be ignored, assuming that time out probabilities are roughly equivalent across all aiming locations.

**References**

Rousseeuw, P. J., & Driessen, K. V. (1999). A fast algorithm for the minimum covariance determinant estimator. *Technometrics*, *41*(3), 212–223.

Verboven, S., & Hubert, M. (2005). LIBRA: a MATLAB library for robust analysis. *Chemometrics and Intelligent Laboratory Systems*, *75*(2), 127–136. doi:10.1016/j.chemolab.2004.06.003
